# The experiences of mothers of children and young people with intellectual disabilities during the first COVID‐19 lockdown period

**DOI:** 10.1111/jar.12884

**Published:** 2021-03-23

**Authors:** Gemma Rogers, Gisela Perez‐Olivas, Biza Stenfert Kroese, Varsha Patel, Glynis Murphy, John Rose, Vivien Cooper, Peter E. Langdon, Steve Hiles, Clair Clifford, Paul Willner

**Affiliations:** ^1^ The Tarentfort Centre Kent and Medway NHS and Social Care Partnership Trust Dartford UK; ^2^ Division of Psychiatry University College London London UK; ^3^ Hertfordshire Partnership University NHS Foundation Trust St Albans UK; ^4^ School of Psychology University of Birmingham Birmingham UK; ^5^ Birmingham Community Healthcare NHS Foundation Trust Birmingham UK; ^6^ Tizard Centre University of Kent Canterbury UK; ^7^ Challenging Behaviour Foundation Chatham UK; ^8^ Centre for Educational Development, Appraisal and Research University of Warwick Coventry UK; ^9^ Centre for Mental Health and Wellbeing Research Warwick Medical School University of Warwick Coventry UK; ^10^ Coventry and Warwickshire Partnership NHS Trust Coventry UK; ^11^ Swansea Trials Unit Clinical Research Facility Institute of Life Science Swansea University Swansea UK

**Keywords:** caring responsibility, children, Covid‐19, intellectual disabilities, mothers, young people

## Abstract

**Background:**

Recent COVID‐19 lockdown restrictions resulted in reduced access to educational, professional and social support systems for children with intellectual disabilities and their carers.

**Aim:**

The aim of this study was to gain insight into the ways mothers of children with intellectual disabilities coped during the first 2020 lockdown period.

**Methods:**

Eight mothers of children with intellectual disabilities were interviewed. The recordings of these interviews were subjected to a thematic analysis.

**Results:**

Three main themes were identified: carrying the burden; a time of stress; and embracing change and looking to the future.

**Conclusions:**

All mothers experienced increased burden and stress. However, some also described some positive impact of lockdown conditions on them as well as on their child's well‐being and behaviour. These findings are discussed in the light of the (Journal of Applied Research in Intellectual Disabilities, 33, 2020, 1523) survey results on parental coping and suggestions for future service provision during pandemic conditions are proposed.

## 1 INTRODUCTION

The first lockdown during the COVID‐19 pandemic in England involved measures of social distancing, social isolation and staff redeployment from mental health and disability services. This resulted in reduced access to educational institutions and medical and social care for those with intellectual disabilities and their carers at a time of enhanced need (UK Parliament, 2020; Cullen et al., 2020; Gulati et al., 2020). Several news stories published in the early weeks of lockdown, when access to professional support and services such as respite were much reduced (Mind, 2020), corroborate how difficult it was for family carers of those with intellectual disabilities. Some reported being on the brink of collapse, at breaking point and forgotten and ignored (Couglan, 2020; Hill, 2020; Youssef, 2020). In addition, a short report using mixed‐methods highlights that many UK families with children with special education needs and disabilities (SEND) had a wide variety of unmet support needs during the initial phase of lockdown (Toseeb et al., 2020). Families of children and young people with autistic spectrum disorder in the UK also reported that their family needs had not been adequately addressed. Although more than half of these families had access to at least one type of specialist support, this was not always timely or sufficient (Pavlopoulou et al., 2020).

Family and other unpaid and informal carers play a key role in caregiving for individuals with intellectual disabilities (Griffith & Hastings, 2014; Mansell & Wilson, 2010), and the COVID‐19 pandemic has led to a heavy reliance on informal home care provision. Insight into the challenges informal carers are currently facing is still limited (Chan et al., 2020). A substantial body of evidence highlights the difficulties that family carers experience in ‘normal’ times when supporting a child with intellectual disabilities including experiences of depression, stress and carer burden and the need they have of a supportive environment, particularly if economically disadvantaged (Hastings, 2002; Resch et al., 2012; Willner & Goldstein, 2001). A recent study asking parents of children with SEND in the UK how COVID‐19 affected their own mental health and that of their child found that both parents and children experienced loss (loss of routine, loss of support network and structures, loss of specialist input and, for a minority, financial loss), worry and changes in mood and behaviour, with some parents reporting feeling overwhelmed with the new demands placed on them without extra support or respite (Asbury et al., 2020).

Social restrictions implemented to manage the first‐wave and subsequent peaks of COVID‐19 have the potential to threaten the mental health not just of family carers but also of their children with intellectual disabilities (Brooks et al., 2020; Fegert et al., 2020) which is likely to lead to an increase in challenging behaviours (Alexander et al., 2020; Courtenay & Perera, 2020), adding to caregivers’ stress. An online parental survey found that children with an autism spectrum disorder who are highly reliant on established relationships and routines experienced a worsening of behaviour problems when their daily routines were disrupted during the lockdown period (Colizzi et al., 2020). However, anecdotally, a minority of families reported that COVID‐19 had had little impact on mental health or even led to improvements for children who struggled at school and felt safest at home (Asbury et al., 2020), suggesting that the predictability of their life under lockdown lead to a reduction in challenging behaviours (Rose et al., 2020).

The high prevalence of mental and physical health multi‐morbidity in people with intellectual disabilities (Kinnear et al., 2018) is likely to heighten many carers’ worries about the health of those they care for during the pandemic (Gulati et al., 2020). Data from the 2017–2018 influenza epidemic underscore the increased risk of illness for those with intellectual disabilities (Cuypers et al., 2020), and the COVID‐19 pandemic has therefore left many informal carers with extra responsibilities. In many cases, they had to deal with the potential contamination of their homes and with increased demands of care in the absence of school or day‐service provision (Coyne et al., 2020). In June 2020, Carers Trust Scotland, 2020, surveyed 214 young carers aged 12 to 25 providing unpaid care at home for family/friends. These young carers reported that since the pandemic their mental health had worsened, they felt less connected to others and had extra caring responsibilities and worries about the future. Willner et al. (2020) also concluded that the strict lockdown during the first COVID‐19 peak had a detrimental effect on the mental health of parents of children with intellectual disabilities.

The longer the hours of care dedicated to a child with intellectual disabilities and the higher their level of dependency, the higher the levels of strain experienced by the carer (Tsai & Wang, 2009). In addition, financial concerns and social isolation also contribute to caregivers’ perceived burden of care and well‐being (Resch et al., 2012; Thompson et al., 2014). Poor social support and challenging behaviours in children with intellectual disabilities are associated with increased psychological morbidity and distress in parents (Gallagher et al., 2008; Rose et al., 2016) yet it was found that during the first lockdown social support was poorest for carers dealing with the most challenging behaviours and who had least resources (Willner et al., 2020).

There is no doubt that the COVID‐19 pandemic triggered significant changes in the lives of many informal carers of children with intellectual disabilities and the impact of those changes is yet to be fully determined (Courtenay & Perera, 2020). The concern of many families is that people with intellectual disabilities may be forgotten as the pandemic unfolds (Silverman, 2020). It is therefore important to gain insight into the ways informal carers coped during the first lockdown period to better anticipate their needs as further restrictions are required. As part of a larger survey study (Willner et al., 2020), which looked at the impact of lockdown on carers of children and adults with intellectual disabilities mental health compared with parents of children without disabilities, we conducted qualitative interviews with a small subsample of informal carers of children with intellectual disabilities to explore their narratives in order to gain in‐depth knowledge of what they found challenging and what they found useful when the first lockdown measures were in place.

## 2 METHOD

This study received a favourable ethical opinion from Swansea University Dept. of Psychology ethics committee [ref. 3874].

### 2.1 Participants

Eight carers of children with intellectual disabilities were recruited and interviewed. For inclusion in this study participants required to (1) be aged 18 or over, (2) live in the UK, (3) have access to the Internet and comfortable with answering questions over the phone or using video conferencing, (4) be the primary care provider for a child with intellectual disabilities (<18 years). See Table 1 for participants’ demographic details.

**TABLE 1:** Demographic details of carers and their children with Intellectual Disabilities

Participant number	Child gender	Degree of intellectual disabilities	Degree of autism spectrum condition	Degree of challenging behaviour	Child's age (years)	Carer's relationship to the child with intellectual disabilities
C01	M	Severe	Moderate	Severe	17	Mother
C02^a^	M	Severe	Severe	Moderate	10	Mother
C03	M	Mild	Severe	Severe	13	Mother
C04	M	Mild	Mild	Moderate	14	Mother
C05	M	Moderate	Moderate	Mild	13	Mother
C06	M	Severe	Severe	Moderate	4	Mother
C07	M	Severe	Severe	Moderate	16	Mother
C08	F	Moderate	N/A	Mild	15	Mother

^a^ Participant C02 refers to two children during interview. However, in the main survey study (Willner et al., 2020), they only provided data for the child whose details are included above.

### 2.2 Procedure

**FIGURE 1:**
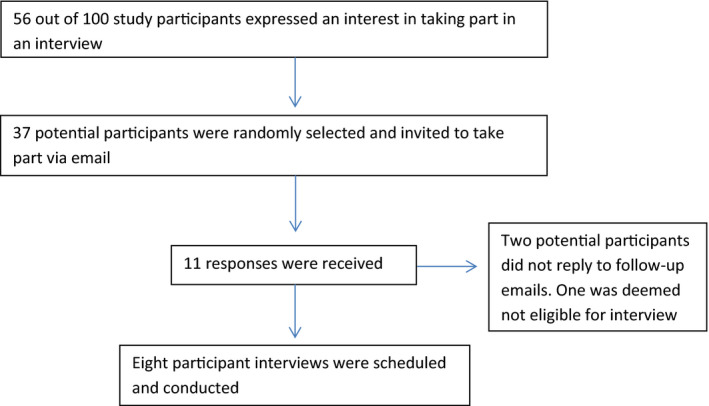
Participant recruitment procedure

Carers of children with intellectual disabilities (*n* = 100) who completed an online survey presented via the RedCap online platform as part of a larger study (Willner et al., 2020) were invited to participate in an interview. From those that expressed an interest in being interviewed (*n* = 56), 37 were selected at random by a member of the research team and invited to take part (Figure 1). In addition to the consent already obtained for taking part in the main study (Willner et al., 2020), participants gave their verbal consent for this study after being read the information sheet by the researcher, this detailed what their participation entailed.

Response and recruitment details are presented in Figure 1 below.

Semi‐structured interviews were conducted by a member of the research team during the months of May and June 2020, when a strict national lockdown regime was in place. The interview schedule is presented in Table 2. Prior to interviewing, the schedule was practised to ensure consistent delivery. Interviews were not conducted face to face due to the lockdown restrictions. Seven interviews were recorded telephone calls as per carers’ preference, and one interview was a recorded video call using the platform Zoom. The median interview time was 22.

**TABLE 2:** Interview schedule (prompts in italics were not asked if the participant had already addressed the topic)

Can you tell me about what it is normally like to care for ……? *What are the challenges?* *What are the rewards?* *What services do you normally receive? (school*, *day services*, *respite* / *short breaks)* *What support do you normally get? (family*, *friends*, *etc*.*)* How have things changed for you since the COVID−19 social isolation/distancing? *What things have stopped or changed? (services but also support from social networks)* *How has daily life changed for you?* *What have you found most challenging? (prompt for examples)* *How does it affect you? (e*.*g*. *health*, *mood*, *sleep*, *routines and relationships)* *How does it affect the person you care for?* *How does it affect the other people who live in your house?* *Do you think your caring situation is understood by others?* What support do you have at the moment? *Others in the household?* *Professional support?* *Friends and family?* *Ask about types of contact*, *phone*, *email*, *Skype*, *Facebook*, *other social media*, *parent support groups*, *etc*. *How do you get information—do you have someone to contact if you need help?* Is there anything that you don't have at the moment that would help you manage better? *Ideas for other support?* *Lessons learnt (looking back*, *etc*.*)?* *What are you main concerns right now?* What helps you most to cope with the current crisis? *Do you have any tips for other carers?* *Any changes that you will keep going when COVID*−*19 social isolation*/*distancing finishes?*

### 2.3 Analysis

The Halcomb and Davidson (2006) method was used for data analysis which does not require transcription of the interview recordings. This method is argued to be a valid and cost‐effective means of data management, particularly suitable for mixed‐method investigations. It involves making field notes during interviews, reviewing them and carrying out a content analysis to identify themes and sub‐themes. The interviews were conducted by GR who also undertook the content analysis using NVivo, a qualitative data analysis software package which helps organise and structure findings within the data.

The interviews were not transcribed for reasons described by Halcomb and Davidson (2006). The following steps of analysis were conducted.

- Step 1: Audio taping of interviews and concurrent note taking
- Step 2: Reflective journaling immediately post‐interview
- Step 3: Listening to the audiotape and amending/revision of field notes and observations
- Step 4: Preliminary content analysis
- Step 5: Secondary content analysis
- Step 6: Thematic review

Good inter‐rater agreement was established by means of another author (VP) also analysing one of the interviews, and GR and VP discussing the themes identified with two of the other authors (BSK and GPO). BSK supervised the analysis throughout, and the final themes were discussed and agreed with four of the authors (GR, GPO, VP and BSK).

## 3 FINDINGS

Three main themes were identified: (1) Carrying the burden; (2) A time of stress; and (3) Embracing change and looking to the future. Each of these themes comprised three subthemes (Tables 3 and 4).

**TABLE 3:** Reasons for using the Halcomb and Davidson (2006) method

The Halcomb and Davidson (2006) method was adopted as it has the following advantages: The costs associated with transcription (time, physical and human resources) are significant. The process of transcription is open to a range of human errors, including misinterpretation of content, cultural differences and language errors. The use of field notes taken during an interview and immediately afterwards has been found to be superior to the exclusive use of audio recordings that are subsequently verbatim transcribed. Field notes capture researchers’ thoughts and interpretations during the process of listening to audio recordings. Audio recordings can be beneficial in assisting interviewers to fill in blank spaces in their field notes and check the relationship between the notes and the actual responses. This can reduce interviewer bias. Audio recordings allow supervisors certify that data reported by a researcher are true and accurate. Where there is ambiguity of meaning, the audio recording can clarify the intended meaning from the original source. Using the original recording of the conversation allows researchers to recreate the nuances of the conversation, such as voice, tone and the specific language of participants, which may assist in more complex analysis.

**TABLE 4:** Themes, sub‐ themes and participants contributing

Themes	Participants contributing	Subthemes	Participants contributing
Carrying the burden	C01, C02, C03, C04, C05, C06, C07, C08.	*Abandoned*	C01, C02, C05, C07, C08.
		*Carer mental health*	C01,C03, C04, C05, C06, C07, C08.
		*Stigma*	C03, C05, C06, C07.
A time of stress	C01, C02, C03, C04, C05, C07, C08.	*Fear*	C01, C02, C03, C08.
		*No break*	C03, C05, C07, C08.
		*Powerlessness*	C01, C02, C03, C04, C05, C07, C08.
Embracing change and looking to the future	C03, C04, C05, C06, C07, C08.	*Less pressure*	C04, C06, C07, C08.
		*‘Foot off the gas’ – now and in the future*	C06, C07, C08.
		*Resilience*	C03, C05, C07, C08.

### 3.1 Theme 1—Carrying the burden

This theme describes the extra work and responsibilities the mothers were required to take on during the lockdown period. For the majority of participants, services and support systems previously in place for children with intellectual disabilities and their carers were withdrawn during lockdown restrictions. This put pressure on carers to develop new ways of managing caring responsibilities:

He needs lots of sensory stimulation that has become more difficult during lockdown because I can’t offer him much variety. We used to go out and do lots of different things but obviously we can’t and so the challenging behaviour has increased, a lot of old habits have come back, it’s his way of communicating that he’s frustrated and that he’s not really getting what he needs. (Participant C01)

A narrative of doubt and guilt runs through most of the interviews e.g. *Am I a bad mum?* (Participant C06). These feelings were sometimes intensified by exposure to social media:

I see these mums on Facebook doing all these fabulous things, and I think I’m just breathing and getting through the day. (Participant C03)

Mothers reported difficulty in providing the level of support for their children that they received prior to lockdown typically offered by external resources such as school, social services, respite, additional carers, family and friends. Most of these fell away when lockdown measures were implemented which evoked feelings of despondency:

I feel he is missing out as he’s not able to access learning in the way he did in the school environment. There is an emotional aspect to that, I feel guilty… he has a lot of unstructured hours at the moment where he would be better served doing something else… I get that constant nagging feeling. (Participant C07)

It has had a huge effect [lockdown]; it’s brought back these behaviours like making himself vomit for sensory stimulation. He is in nappies day and night and if I don’t notice him soon enough he will smear. There’s also lots of scratching, biting, and pulling hair and clothes. (Participant C01)

For all participants, the additional caring responsibility imposed due to lockdown resulted in personal sacrifices being made in order to maintain a level of support necessary for the needs of the child they care for:

We can’t work because we just can’t dedicate the time to working… the children have full time needs. (Participant C02)

Some mothers were offered support, for example friends or family offering to deliver food supplies. However, some of these offers were declined due to participants perceiving these tasks as their own responsibility and not wanting to impose on to others, feeling as if they, alone had to carry the burden of caring.

#### 3.1.1 Abandoned

For some, pre‐lockdown caring responsibilities had already impacted on their relationships with others: *I don't have relationships due to my circumstances* (Participant C01), and access to professional services had always been the main source of support. However, as national restrictions were imposed and access to these services ceased, all participants reported feeling abandoned by professional services. One mother felt she had become *an afterthought* (Participant C02):

The way I would describe the current situation, I feel abandoned. The summer holidays would usually be deemed a challenging time for families like us but we would have support, and yet this has gone on a lot longer with no support…we are told to believe this is hard for everyone, we are all in the same boat, well we’re not. (Participant C07)

Most mothers felt that the burden of care had been *pushed back to us families with no support*. (Participant C07).

#### 3.1.2 Carer mental health

The burden of care, for some, impacted on their own mental health, with reports of *less and less interest in anything* (Participant C04), withdrawal, stress, hopelessness, and needing to increase prescribed medication:

We’ve had our medication increased [by the GP]; we haven’t had access to the things that help our mental health [talking therapies etc.]… It’s very much like living in a PTSD environment…you are always waiting to for an attack… (Participant C02)

#### 3.1.3 Stigma

Some mothers referred to themselves as *overlooked* (Participant C02) or *a marginalised part of the community* (Participant C02) and had experienced stigmatisation pre‐Covid due to their child's disabilities. However, during the period of lockdown, they reported an increase in negative attitudes towards their child:

The amount of looks people have given him when we’ve drove somewhere. People have been like ‘you’re not from here’, and we say ‘he’s got a disability’, and they say ‘he doesn’t look like he does’, they don’t understand. It’s the first time since he’s been born I’ve had people shout at me about him. (Participant C06)

### 3.2 Theme 2—A time of stress

This theme is about the mother's subjective experiences caused by the extra burden imposed upon them as carers during the lockdown period. The additional demands of caring during this period resulted in increased levels of stress.

#### 3.2.1 Fear

For most participants, lockdown was experienced as living in a state of fear, *on a knife's edge* (Participant C05). Participants reported navigating an unpredictable, inescapable home environment, sometimes being *led into a false sense of security* (Participant C08), only to experience further crises.

Home is not a safe place for us as she can lash out at us. Whereas if you’re in the car listening to the wheels on the bus for the eighth time she’s happy, we are safe. But then we had the anxiety of are we going to be stopped and accosted for being away from home? (Participant C02)

One mother described her experience of her two children with severe autism receiving a COVID‐19 test at a test site. The event was particularly distressing for the children because of sensory overload and fear induced by the sight of soldiers at the test site.

My son was fixated on the soldiers… he was saying ‘go home, go home’… he did the test but was very scared about what that means, why the soldiers were there. My daughter was very distressed which we knew she would sensory wise… we had to physically pin her down and we did get attacked – scratching, pinching, it’s been quite distressing. (Participant C02)

#### 3.2.2 No break

One of the biggest challenges reported by participants was having no respite from their caring responsibilities. For some, previously being able to go to a place of work was considered a break, but lockdown and working from home made this impossible. The demand of caring left these mothers feeling *physically drained from not having a break week on weeks on weeks*. (Participant C07).

#### 3.2.3 Powerlessness

The continuous threat of challenging behaviour in a confined space induced a sense of powerlessness and *surrender* (Participant C08), with one mother describing …*knowing when to pick my battles* (Participant C04). For some, an uncertain future due to COVID‐19 became overwhelming especially for those caring for children who struggle with change and a lack of routine:

The fact there is no certainty, that vague voidness of not knowing is difficult for him and the lack of concreteness. (Participant C07)

### 3.3 Theme 3—Embracing change and looking to the future

In contrast to the severely challenging experiences described above, this theme concerns some of the mothers’ positive experiences, directly related to the lockdown conditions.

#### 3.3.1 Less pressure

The elimination of many daily pressures (e.g. getting ready for school) resulted in a reduction in some children's challenging behaviours. Improvements were also reported in mood, sleep, seizures, obsessive and compulsive routines, speech, and a number of children generally appeared more relaxed. Participants attributed these improvements to the removal of task demands:

She wasn’t her usual self, she was really happy all the time because I wasn’t asking anything of her. (Participant C08)

Moreover, some mothers reported experiencing a reduction in their own stress levels during lockdown, which they associated with having more time:

I was a lot more stress free because we had nowhere to go so I was able to give her more time. (Participant C08)

### 3.4 ‘Foot off the gas’—now and in the future

A number of mothers reported that concerns related to the child's well‐being when away from home (e.g. the child having a seizure at school) was eased and considered better managed at home:

I’m loving it more because I am working from home, more relaxing because I’m not watching my phone, expecting the school to call because something has happened. I’m getting more work done because I’m not worrying about him so I can focus on my work; he is such a different person he is happy, he’s learning. (Participant C03)

Lockdown ensured some households spent significantly more time in each other's company. This was considered to benefit the entire household and participants reflected on how they wanted to hold on to these positives:

I need to not do as much work maybe take a step back, I’ve really enjoyed this time when everyone has been together and happy. (Participant C08)

We have been going for walks together; he said going out for walks makes his autism ‘fresher’, he’s finding it beneficial. (Participant C05)

For some, lockdown became a period of reflection, time to *take a step back* (Participant C08). This provided an opportunity to consider how things may be done in the future:

Life has calmed down considerably we were on the go a lot and now we’re not so that’s a positive thing. We’ve had more family time which helps so actually although it’s been quite an anxious time, from a family point of view we’ve benefited massively … going forward I won’t take work as seriously I won’t put as much clubs in and more family time. (Participant C05)

### 3.5 Resilience

Participants were able to reflect on their resilience during this difficult time:

Participant C07 reasoned that future school holiday periods (previously considered challenging) would not seem so daunting, given their proven ability to *get through* (Participant C07) the lockdown period. In the face of the burden of caring recently experienced, she elaborated further:

If you can come through this you can do anything… previously I would have felt a bit overwhelmed and out of control, but now I am thinking about that, laughing at reflecting back on it, it puts it into perspective. (Participant C07)

Some reported developing effective coping strategies to combat depression, anxiety and feeling powerless by developing exercise routines or hobbies:

I run, I exercise or do my cross stitching. (Participant C06)

## 4 DISCUSSION

This study is one of the first to explore the early experiences of lockdown of mothers of children with intellectual disabilities, focusing on the impact of social restrictions on their well‐being. The eight mothers who took part in this qualitative study gave detailed and vivid accounts of their experiences and reported increased burden and stress during the months of May and June 2020 when measures of social restriction were in place and access to professional support and services was limited. Their accounts highlight their difficulties in managing daily activities during the COVID‐19 outbreak as their children with intellectual disabilities presented challenging behaviours and education, healthcare and respite services, and other professional support was limited or not available. Their accounts echo those reported by other researchers (Asbury et al., 2020; Colizzi et al., 2020; Toseeb et al., 2020).

However, it is important to note that in spite of these difficulties, most of these mothers also touched upon positive experiences such as reduced daily pressure and more opportunities for spending time as a family, and enjoying hobbies and leisure activities. They included mothers of children with severe intellectual disabilities and/or Autism Spectrum Condition with severe or moderate challenging behaviour, suggesting that the mothers’ ability to cope and embrace change may not be determined solely by the child's level of disability and/or challenging behaviour. This is in line with Resch's model (2012) of well‐being for parents of children with intellectual disabilities which posits that feeling included and accepted in the surrounding social environment has a greater impact on parents’ well‐being than the severity of the child's disabilities. It is therefore of concern that family carers of those with the greatest needs and most severe challenging behaviour reported least social support from their immediate environment during lockdown (Willner et al., 2020).

Our analysis contextualises Willner et al. (2020) findings; the withdrawal of services and support systems that were in place for mothers of children with intellectual disabilities prior to lockdown in addition to the social restrictions placed on them created additional caring responsibilities for them. They describe the difficulties of juggling home schooling and work and how lockdown increased their child's challenging behaviours, contributing in turn to their burden of care and mental health problems.

Some mothers in our study described how they were offered but declined support from family and friends as they did not want to impose on others and because they perceived their caring tasks as their own responsibility. These descriptions complement research that found that individuals with depression were reluctant to seek help from family and friends as they were concerned about what others would think, ashamed about others knowing of their problems, and not wanting to be a burden (Griffiths et al., 2011). Mothers of children with intellectual disabilities who perceive high levels of stigma have been found to interact less frequently with their peers in informal settings of homes and neighbourhoods (Green, 2003). Narratives of doubt, guilt and despondency are prevalent in our sample and confirm findings of our quantitative study (Willner et al., 2020) that these negative cognitions and feelings underscore the mental health problems experienced by informal carers of children with intellectual disabilities. The subtheme of powerlessness, characterised by surrender in the face of continuous threat of challenging behaviours and an uncertain future, sheds light on the sense of hopelessness of the caregivers captured in our quantitative study by the defeat/entrapment scale (Willner et al., 2020). The lack of support from services, family and the community for those who need it the most had a significant negative impact on the mothers we interviewed. They reported feeling abandoned, stigmatised and experienced an increase in mental health problems during the first lockdown period.

All of the mothers in our study experienced lockdown as a time of stress. They reported living in a state of fear, navigating an unpredictable, inescapable home environment. However, they were also able to identify resilience in their response to the situation. This supports Resch's model (2012) which describes how parents recognise potential harm to themselves resulting from the challenges and responsibilities of raising a child with disabilities as well as identifying positive personal growth, whereby belief in new possibilities, personal strength and appreciation of life contribute to their well‐being.

Anecdotal evidence of how the first lockdown period may have contributed to the reduction of challenging behaviours of some children with intellectual disabilities (Rose et al., 2020) is provided by the narratives of six of the eight mothers interviewed. Their accounts shed light on how the absence of daily pressures had a positive impact on their child's mood and behaviour. Bentenuto et al. (2021), Pavlopoulou et al. (2020) and Asbury et al. (2020) also report benefits for families due to the loosening of daily routines. This suggests that more time to spend with the child, building low arousal routines at home and appropriate educational activities set by the school (as proposed by Toseeb et al., 2020) may strengthen the parent–child relationship and contribute to the well‐being of at least some children with intellectual disabilities. The subthemes ‘foot off the gas’ and ‘resilience’ with descriptions of enjoying time with the family and developing ways to cope further validate Resch et al. (2012) model which describes how appraisal of threats but also opportunities for growth can co‐occur in parents caring for children with intellectual disabilities.

There are several limitations to the study. We recruited a sample of mothers with caring responsibilities for a child with significant and relatively complex needs. They were also a relatively affluent sample (Willner et al., 2020) and this may account for the fact that financial pressures affecting their well‐being were not voiced during any of the interviews. Greater care needs of informal carers in relation to financial pressures during the first national lockdown were found detrimental to their mental health (Willner et al., 2020). Thus, it is important to also explore the experiences of informal carers from economically disadvantaged backgrounds as the pandemic continues. Moreover, our findings are based on a small (*n* = 8) sample and the views and experiences of those who chose not to take part may be different. There may also be a sampling bias towards those mothers who were most able to cope during the lockdown restrictions. Alternative routes for recruitment, for example contacting local self‐advocacy groups, should be considered for future studies. Including the voices of the fathers, the children with intellectual disabilities and their siblings could also shed further light on the struggles these families experience when social restrictions are reinstated. There are many reasons to further investigate how different ethnic groups experience lockdown conditions, not least because they have varying levels of susceptibility to COVID‐19 (Otu et al., 2020).

The current study combined the voices of mothers of primary and secondary school‐age children and future research may want to hone in on these groups separately in order to identify possible differences between their experiences. At the time of writing, a second national lockdown is in place, leaving people with intellectual disabilities and their families feeling that they are in a perpetual cycle of lockdown (Evans, 2020). Following up on their experiences would provide further valuable insights into the long‐term difficulties these families are facing.

A strength of the study is its qualitative nature by which the voices of mother of children with intellectual disabilities can be heard. This is to our knowledge the first study that explores the narratives of mothers of children with intellectual disabilities by means of a variety of open‐ended questions that tap into how social distancing and isolation have affected their well‐being and that of their children. Because the interviews took place in May and June 2020 and it was only from 15th June onwards that the first lockdown restrictions were gradually eased, it is fair to assume that their experiences were fresh in their mind and allowed them to provide us with rich and detailed data.

Some of our findings may be used to inform future service responses to these families amidst the COVID‐19 crisis, for example, by having a care plan in place based on the carers’ and children's needs and preferences (Alexander et al., 2020). Some elements of the care plan could include local online support groups or helplines, respite options, remote professional advice and ensuring that children have easy access to education tailored to their needs. To make sure that families with greater care needs feel socially included and supported, neighbourhood schemes could be developed. During the first lockdown, neighbours were encouraged to support older people (Age UK, 2020) and similar support for families caring for children with intellectual disabilities could be encouraged through national and local media. Community education targeting de‐stigmatisation could also be of benefit.

The use of IT was not raised by the mothers in this sample although this topic was discussed by participants of its sister study of family carers of adults with intellectual disabilities, who mentioned it as a useful source of support for keeping in touch with friends and family and other parent‐carers of adults with intellectual disabilities (Patel et al.,, in preparation). Patel et al's findings are consistent with a review highlighting the usefulness of Internet‐based support groups for carers of adults with intellectual disabilities as a way of keeping socially engaged (Perkins & LaMartin, 2012). For parents of children of school age, IT will have further relevance in terms of home schooling and communicating with educational and other professionals involved in their child's care (Lee et al., 2020).

Finally, the voices of children with intellectual disabilities and their families need to be continuously listened to as the pandemic unfolds in order to become more sensitive to their needs by means of care plans that make them feel included, appreciated and respected, instead of overlooked and marginalised.
